# Pediatric ultrasound-guided dorsal penile nerve block and sedation in spontaneous breathing: a prospective observational study

**DOI:** 10.3389/fmed.2023.1268594

**Published:** 2023-12-04

**Authors:** Bruno Dottore, Francesco Meroi, Serena Tomasino, Daniele Orso, Matteo Comuzzi, Nicola Vernaccini, Luigi Vetrugno, Sergio Intini, Tiziana Bove

**Affiliations:** ^1^Azienda Sanitaria Integrata Friuli Centrale (ASUFC), Hospital of Palmanova, Italy Anesthesia and Intensive Care Service, Palmanova, Italy; ^2^Department of Anesthesia and Intensive Care, University-Hospital of Udine, Udine, Italy; ^3^Department of Medicine, University of Udine, Anesthesia and Intensive Care Clinic, Udine, Italy; ^4^Department of General Surgery, University-Hospital of Udine, Udine, Italy; ^5^Department of Medical, Oral and Biotechnological Sciences, University of Chieti-Pescara, Chieti, Italy; ^6^Department of Anesthesia and Intensive Care, University of Chieti-Pescara, Chieti, Italy; ^7^Department of Medicine, University of Udine, General Surgery Clinic, Udine, Italy

**Keywords:** pediatric, ultrasound, dorsal penile nerve block, circumcision, spontaneous breathing, mechanical ventilation, laryngeal mask, sedation

## Abstract

**Background:**

Worldwide, one of the most common surgical procedures in the pediatric population is circumcision. There is no consent on the best anesthesiologic approach. This study aimed to investigate ultrasound-guided dorsal penile nerve block (DPNB) plus sedation in spontaneous breathing as a time-saving, safe, effective, and opioid-sparing technique.

**Aims:**

The primary outcome was the assessment of the time from the end of surgery and the discharge to the post-anesthesia care unit. Secondary outcomes were to evaluate the cumulative dosages of opioids, differences in pain levels between the two groups, and complications at the awakening, 4 h and 72 h after surgery, respectively.

**Methods:**

This was a prospective study with a retrospective control group, approved by the Friuli–Venezia Giulia Ethics Committee. Children in the intervention group received an ultrasound-guided DPNB under sedation and spontaneous breathing. With the probe positioned transversally at the base of the penis using an in-plane approach with a modified technique, local anesthetic was injected under the deep fascia of the penis.

**Results:**

We recruited 70 children who underwent circumcision at the University Hospital of Udine, Italy, from 1 January 2016 to 1 October 2021: 35 children in the ultrasound-guided DPNB group and 35 children in the control group. Children who received ultrasound-guided DPNB had a statistically significant lower time to discharge from the operating room, did not require mechanical ventilation, maintained spontaneous breathing at all times, received fewer opioids, had lower mean intraoperative arterial pressures, and lower pain levels immediately after surgery.

**Conclusion:**

Ultrasound-guided DPNB associated with sedation and spontaneous breathing is a time-saving, opioid-sparing, safe, and effective strategy for the management of intraoperative and postoperative pain in children undergoing circumcision.

**Clinical trial registration:** ClinicalTrial.gov (NCT04475458, 17 July 2020).

## Introduction

One of the most frequent surgical procedures in the pediatric population is circumcision, following which postoperative pain could be stressful (1). To minimize psychological trauma in children, adequate analgesia during surgery and in the postoperative phase must be achieved (2).

Usually, the most common anesthesiologic approach is combining regional anesthesia techniques such as caudal block, ring block, or dorsal penile nerve block (DPNB) with general anesthesia (GA) (3).

There is no consensus on whether one anesthesiologic technique is better than the other (4).

The DPNB with a landmark technique has been described by several authors with different approaches and permits to localize anatomically the dorsal nerves of the penis and inject a local anesthetic to block the transmission of the nerve impulses providing adequate anesthesia of the penis (5, 6).

With the spared of ultrasounds in regional anesthesia, a novel ultrasound-guided DPNB has also been performed. Sandeman et al. first described this technique with an out-of-plane approach (7). Suleman et al. presented an in-plane approach in order to be able to follow the needle and precisely locate the tip during its transit through the tissues (8).

In a randomized controlled trial, the authors compared the ultrasound DPNB with the landmark technique associated with GA. They found no difference regarding postoperative analgesia needs in the two groups but an increased time to perform the ultrasound-guided nerve block (9).

This study investigates ultrasound-guided DPNB plus sedation in spontaneous breathing as a time-saving, safe, effective, and opioid-sparing technique.

## Methods

### Study protocol

This was a prospective study with a retrospective control group comprising children admitted to the University Hospital of Udine, Italy, from 1 January 2016, to 1 October 2021. The study was conducted according to the principles of the Declaration of Helsinki. The Ethics Committee of Friuli–Venezia Giulia approved the study with the number ID # 3101, 11 February 2020, and the study was registered at ClinicalTrials.gov number ID # NCT04475458, 17 July 2020.

### Study population

Inclusion criteria were as follows: children from 6 months to 17 years of age enlisted for circumcision for medical purposes, with American Society of Anesthesiologists (ASA) score of <3, and an informed written consent signed by the parents.

Exclusion criteria were allergies to local anesthetics and difficult ultrasonographic window.

### Procedures

During the anesthesiologic interview, informed consent to participate in the study was signed by the parents.

All patients were premedicated with oral midazolam 0.5 mg/kg, with a maximum dosage of 10 mg and a venous access was placed. In the operatory room, heart rate (HR), blood pressure (BP), peripheral oxygen saturation (SpO_2_), end-tidal carbon dioxide (EtCO_2_), and body temperature were monitored.

### Ultrasound dorsal penile nerve block group

The patients in the ultrasound group, after sedation with propofol 1–2 mg/kg and in case with fentanyl 1–2 mcg/kg, maintained spontaneous breathing with a nasal cannula and 2–4 L/min of oxygen. With the patient in the supine position, after disinfection of the pubis with povidone-iodine (Braunol 7.5%, B. Braun, Germany), the anesthesiologist performed an ultrasound-guided DPNB with a linear probe (SonoSite, Washington, United States) using a modified technique based on the one described by Suleman (8). The probe was placed transversally at the base of the penis between the shaft and the scrotum. Once the corpora cavernosa had been identified, the dorsal arteries, dorsal nerves, and dorsal vein were located beneath the deep fascia of the penis (Buck’s fascia).

**Figure 1:**
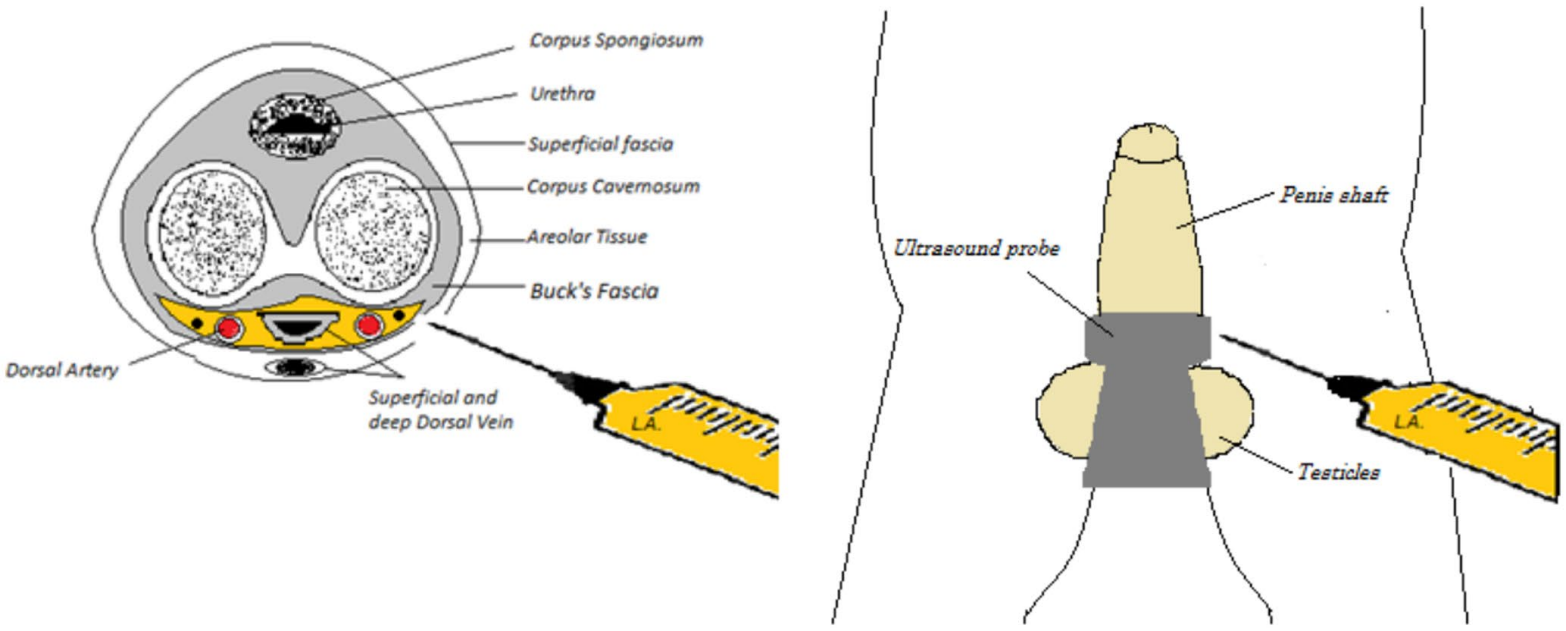
Anatomy of the penis and ultrasound probe position.

**Figure 2:**
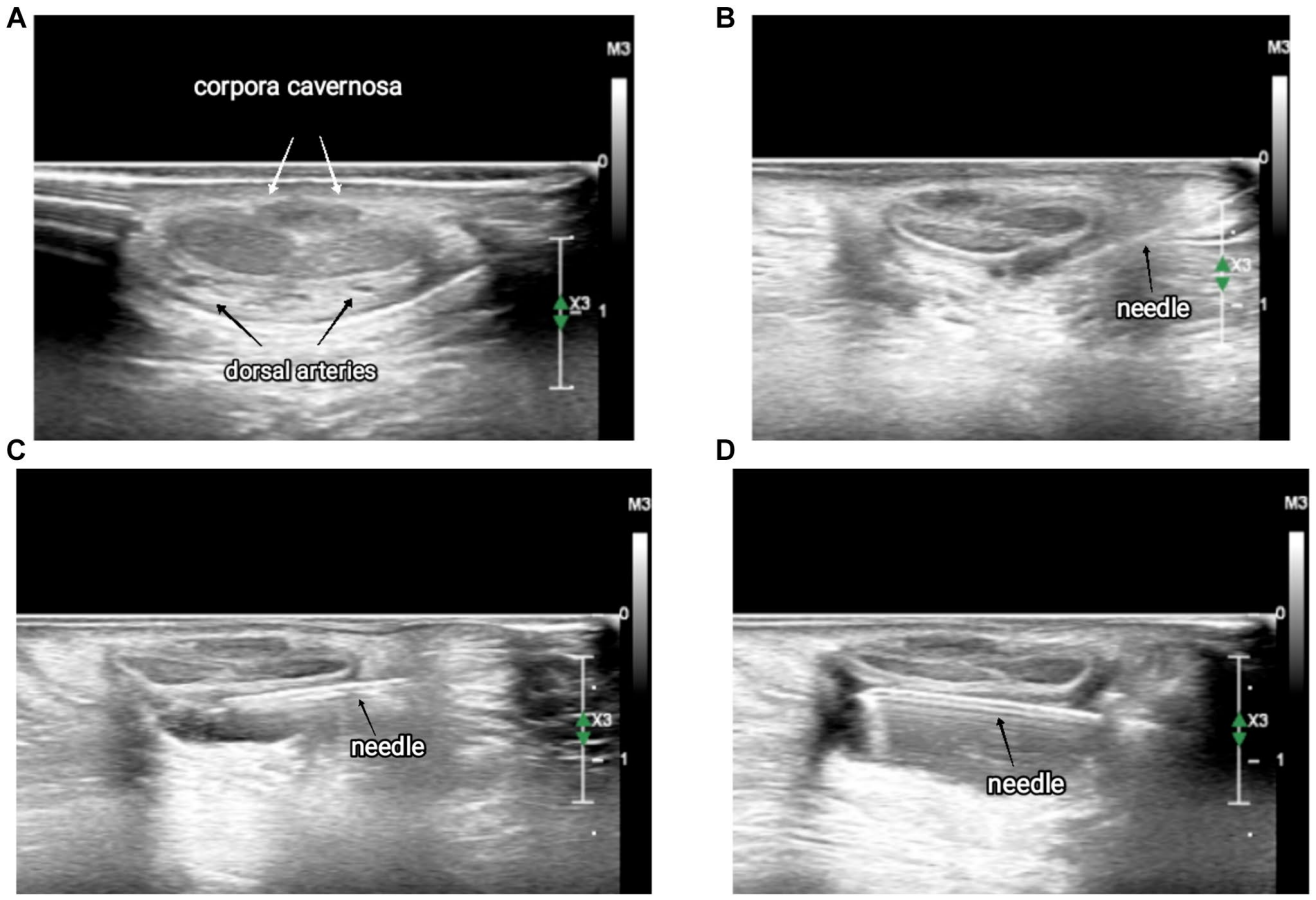
Sequence of ultrasound images of the dorsal penile nerve block. **(A)** Anatomy of the penis, **(B)** local anesthetic injection on the right-hand side, **(C)** local anesthetic injection in the median region, and **(D)** local anesthetic injection on the left-hand side.

With a 50-mm Echoplex+ (Vygon, France) echoic needle, the block was performed with an in-plane approach. After entering from the right-hand side and passing through the skin and the fascia, an aspiration test was performed to avoid intravascular injection and a volume of 0.15–0.25 mL/kg of a mixture of mepivacaine 1% and levobupivacaine 0.25% was injected within the fascia around the dorsal nerves. The needle was then slightly retracted under the corpora cavernosa, and a small amount (1–2 mL) of local anesthetic was deposited on the median line (Figure 1). With continuous ultrasound guidance, the needle was then advanced toward the contralateral nerve, and once under the fascia, the left nerve was blocked as well (Figure 2).

Sedation was maintained with propofol 1% with a syringe pump (DB, Franklin Lakes, United States) set to perform a target control infusion (TCI) with the Paedfusor model and a target plasmatic concentration (Cpt) of 0.6–1.5. Patients maintained spontaneous breathing with oxygen delivered through a nasal cannula and EtCO_2_ monitored.

According to the protocol, if there was an increase in HR or BP of 20% or more, the Cpt of propofol was increased, and if these actions were not sufficient, a GA was performed and mechanical ventilation with a laryngeal mask (LMA) was started.

### Landmark dorsal penile nerve block group

The anesthesiologic management of these patients was different according to the anesthesiologist’s decision. All patients received a GA with an LMA or orotracheal intubation, and the ventilation was set up according to the anesthesiologist’s preference. After anesthesia induction, skin disinfection with povidone-iodine (Braunol 7.5%, B. Braun, Germany), and sterile field preparation, the DPNB was performed by the surgeon using the landmark technique. The block was performed with a syringe and a standard needle using mepivacaine 1%.

The pubis was identified and the injection site was located, after a loss of resistance when the fascia of Scarpa was transfixed by the needle, and the local anesthetic was injected, 4–6 mL bilaterally. 1 or 2 mL of local anesthetic were injected between the penis shaft and the scrotum to cover the ventral part of the glans (5).

### Postoperative multimodal analgesia

During surgery, paracetamol 15 mg/kg was administered to the ultrasound group, while different drugs were used in the control group according to the preferences of the anesthesiologist.

As a rescue therapy after surgery, non-steroidal anti-inflammatory drugs were prescribed and given in the presence of pain.

### Recorded data

Anthropometric parameters, such as age, weight, and height, as well as medical history, ASA status, and clinical conditions on admission, were recorded. Intraoperatively, hemodynamic parameters such as heart rate (HR), peripheral oxygen saturation (SpO_2_), and mean arterial blood pressure (mAP) were measured at the arrival of the patient in the operatory room (T1), induction of anesthesia/sedation (T2), surgical incision (T3), the end of surgery (T4), and awakening (T5). An increase in the HR or BP of 20% or more above the baseline and the necessity to start a GA with an LMA and mechanical ventilation at the skin incision were noted and defined as a failed block. All drugs administered during the intraoperative phase were registered as well as the time from the end of surgery and extubation/LMA removal in the operatory room to the entrance to the post-anesthesia care unit (PACU). Complications at the awakening such as cough, airway obstruction, laryngospasm, bronchospasm, apnea, hypoventilation, and postoperative complications such as nausea, vomiting, penis edema, or ischemia were assessed; 72 h after surgery, patients were evaluated in the surgical ambulatory to detect possible complications such as edema, lesions, infections, and ischemia. Pain was assessed at awakening, 4 h after surgery, and 72 h after surgery. Different pain scales were used according to the age of the patients. The Face, Legs, Activity, Cry, Consolability (FLACC) scale for children under 3 years of age, the Faces Pain Scale for children between 3 and 8 years of age, and the Numeral Rating Score (NRS) scale for children over 8 years of age. Rescue analgesia administered to children was documented.

The Preliminary results of the study were presented as a poster at the European Society of Regional Anaesthesia and Pain Therapy (ESRA) 2020 Annual Congress.

### Study outcome

The primary outcome of this study was to assess the time from the end of surgery and extubation/LMA removal in the operatory room to the awakening of the children and discharge into the PACU.

Secondary outcomes were to evaluate the cumulative dosages of opioids, differences in pain levels between the two groups, and complications at the awakening, 4 h after surgery, and 72 h after surgery.

### Statistical analysis

The distribution between the two study groups was compared with median or mean values (depending on the distribution if normal or non-normal) and with frequency values (for categorical variables). After checking the normality of the distribution through the Shapiro–Wilk test, comparisons were made through the *t*-test or Kruskal–Wallis test (for continuous variables) and the Fisher or chi-square test (for categorical variables).

Multiple pairwise comparisons were made using paired *t*-tests to verify the significance of the differences between the continuous variables repeatedly measured over time.

A value of *p* ≤ 0.05 was considered statistically significant. A Bonferroni method was used for multiplicity correction.

All statistical analyses were performed using open-source software “R: A language and environment for statistical computing,” implementing the “readODS,” “compareGroups,” “multcomp,” “tidyverse,” “rstatix,” and “ggpubr” packages.

The sample size calculated was 70 patients, 35 for each group.

## Results

We included in the study 70 children who underwent circumcision at the University Hospital of Udine, Italy, from 1 January 2016, to 1 October 2021. Thirty-five children were prospectively enrolled, and the anesthesiologist performed an ultrasound-guided DPNB under sedation, while 35 children were retrospectively enrolled as a control group and received GA plus DPNB performed by the surgeon with the landmark technique. All children were ASA score 1 and the two groups were comparable for age, weight, and height. The results are displayed in Table 1.

**Table 1:** Results of the study.

		Population (*n* = 70)	Control group (*n* = 35)	DNPB group (*n* = 35)	*p* value
Demographics					
Age (y)		10 [7–12]	9 [7–11]	11 [7–12]	0.132
Weight (kg)		33.0 [24.2–43.0]	29.0 [24.0–42.5]	35.0 [26.0–44.0]	0.329
Height (cm)		138 [126–151]	137 [124–146]	142 [126–152]	0.194
T1					
HR (bpm)		90 [83–100]	90 [87–102]	89 [81–100]	0.190
mAP (mmHg)		66 [63–72]	68 [65–77]	64 [60–68]	*<0.001*
SpO_2_ (%)		100 [100–100]	100 [100–100]	100 [100–100]	1.000
BR (bpm)		18 [16–22]	18 [17–22]	18 [15–20]	0.189
T2					
HR (bpm)		90 [80–100]	95 [80–105]	88 [79–92]	0.070
mAP (mmHg)		65 [62–71]	68 [63–72]	62 [59–68]	*0.003*
SpO_2_ (%)		100 [100–100]	100 [100–100]	100 [100–100]	1.000
BR (bpm)		18 [15–20]	18 [16–23]	16 [14–20]	*0.040*
T3					
HR (bpm)		88 [81–100]	95 [85–111]	85 [79–93]	*0.014*
mAP (mmHg)		66 [60–70]	68 [65–72]	60 [58–67]	*<0.001*
SpO_2_ (%)		100 [100–100]	100 [100–100]	100 [100–100]	0.558
BR (bpm)		18 [14–20]	18 [16–23]	16 [14–19]	*0.011*
T4					
HR (bpm)		89 [80–98]	90 [85–101]	85 [77–90]	*0.013*
mAP (mmHg)		65 [60–70]	68 [64–72]	60 [58–66]	*<0.001*
SpO_2_ (%)		100 [100–100]	100 [100–100]	100 [100–100]	1.000
BR (bpm)		18 [14–20]	19 [16–21]	16 [14–18]	*0.005*
T5					
HR (bpm)		88 [80–98]	92 [81–104]	84 [78–91]	*0.009*
mAP (mmHg)		66 [62–72]	69 [64–74]	62 [60–66]	*<0.001*
SpO_2_ (%)		100 [100–100]	100 [100–100]	100 [100–100]	1.000
BR (bpm)		18 [16–20]	20 [17–22]	18 [15–20]	*0.023*
Opioids (mcg of fentanyl equivalents)		50.0 [25.0–70.0]	55.0 [50.0–100.0]	30.0 [0–50.0]	*<0.001*
Propofol (mg)		100.0 [70.0–150.0]	100.0 [71.2–150.0]	100.0 [72.5–150.0]	0.583
Rescue dose		0 [0–1]	0 [0–1]	0 [0–1]	0.216
Time to rescue dose (min)		0 [0–60]	0 [0–90]	0 [0–30]	0.197
ΔHR > 20%		8 (11%)	8 (23%)	0	*0.005*
Ventilatory support		35 (50%)	35 (100%)	0	*<0.001*
Flumazenil		0	0	0	1.000
Complications at awakening					0.188
	Deep sedation/difficulty to awaken	10 (14%)	7 (20%)	3 (9%)	
	Shivering	1 (1%)	1 (3%)	0	
Complications at 4 h					1.000
	Minor bleeding	2 (3%)	1 (3%)	1 (3%)	
Complications at 72 h					0.172
	Edematous glans	18 (26%)	12 (34%)	6 (17%)	
	Hematoma	4 (6%)	3 (9%)	1 (3%)	
	Minor bleeding	1 (1%)	0	1 (3%)	
	Grazed glans	2 (3%)	2 (6%)	0	
	Persistent inflammation	3 (4%)	3 (9%)	0	
NRS					
Awakening		0 [0–0]	0 [0–0]	0 [0–0]	*0.006*
4 h		0 [0–0]	0 [0–2.5]	0 [0–0]	0.345
72 h		0 [0–0]	0 [0–0]	0 [0–0]	0.984
Time from end of surgery to discharge in PACU (min)		10 [5–20]	20 [15–27]	5 [2–6]	*<0.001*

HR, heart rate; mAP, mean arterial pressure; SpO2, peripheral oxygen saturation; BR, breathing rate; ΔHR, variation in heart rate. T1, arrival of the patient in the operatory room; T2, induction of anesthesia/sedation; T3, surgical incision; T4, end of surgery; T5, awakening.

**Figure 3:**
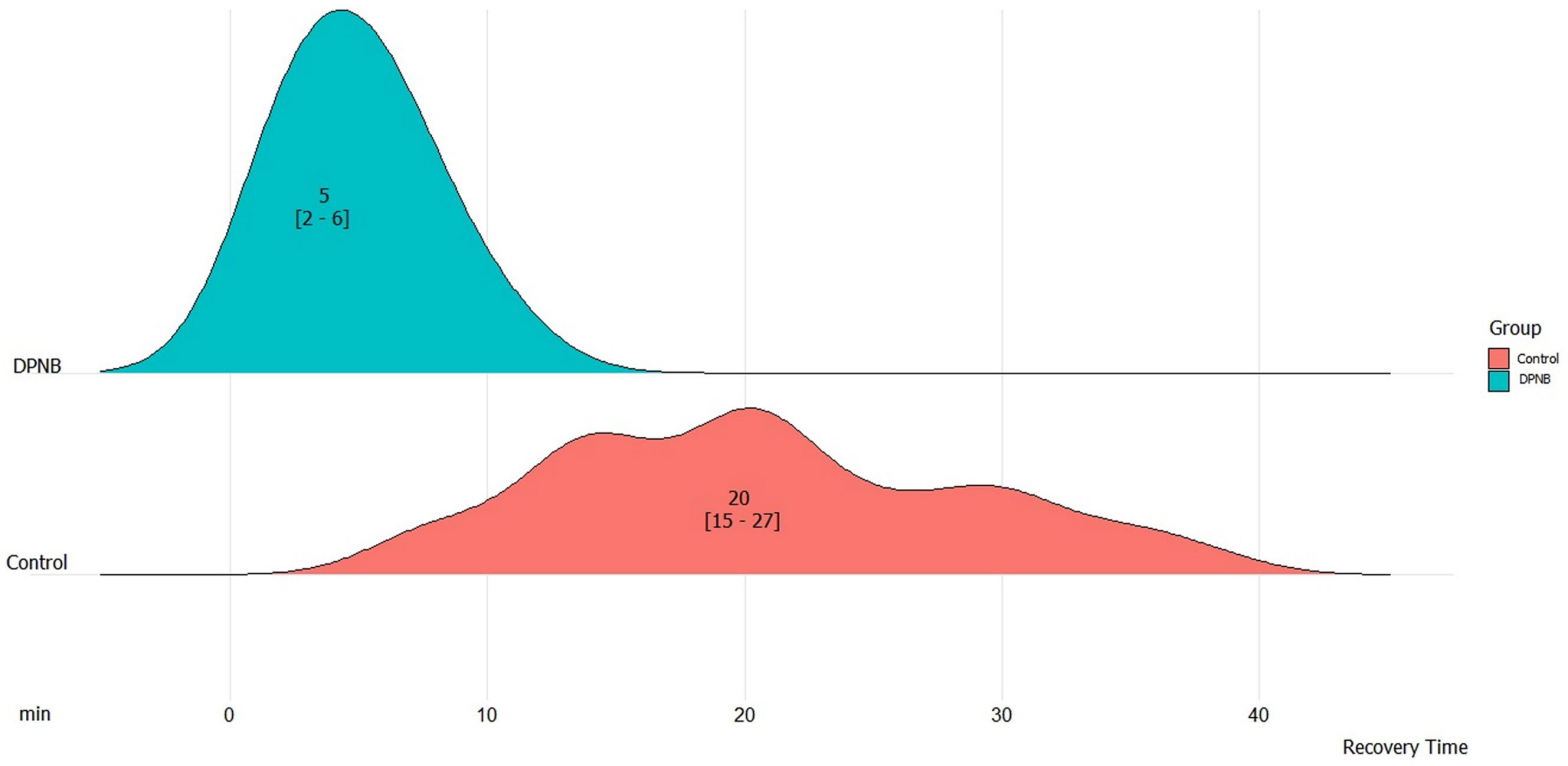
Time to discharge from the end of surgery into the post-anesthesia care unit. DPNB, dorsal penile nerve block.

Children who received ultrasound-guided DPNB had a statistically significant lower time to discharge from the operating room with faster awakening 20.0 min vs. 5.0 min (*p* < 0.001; Figure 3).

All children in the DPNB group did not require mechanical ventilation and always maintained spontaneous breathing during sedation; 8 out of 35 patients (22.9%) of the landmark cohort had a heart rate increase of 20% or more at skin incision, whereas no patient in the ultrasound group did (*p* = 0.005). When compared between the two groups, opioid consumption was statistically significantly lower in the ultrasound-guided population (30 mcg vs. 55 mcg – *p* < 0.001), and mean arterial pressure was also lower. Heart rate was lower in the DPNB at T3, T4, and T5, and respiratory rate was lower at T2, T3, T4, and T5.

Lower pain levels immediately after surgery were evident in the DPNB group (*p* = 0.006), but at 4 and 72 h after surgery, the data suggest a lower rate of children with pain and lower mean pain levels but without statistical significance.

No differences were found between the two groups with regards to complications at 4 h but a lower complications rate, not statistically significant was found at awakening and after 72 h in the DPNB group.

## Discussion

The main finding of this study is that the ultrasound-guided block of the dorsal nerves of the penis combined with sedation and spontaneous breathing is a safe technique that permits saving time with rapid awakening and discharge from the operating room, and reduces opioids consumption in children undergoing circumcision.

Regional anesthesia associated with sedation permits to reach an optimal level of intraoperative and postoperative analgesia, minimizing possible complications associated with GA and airway management in children (10, 11).

Many authors have described the DPNB in the literature. In 1975, Bateman first described the landmark technique followed by other alternative approaches (5).

An ultrasound-guided DPNB was described by Sandeman and Dilley. The probe was placed transversally at the base of the pubic symphysis, and with an out-of-plane approach, the local anesthetic was injected laterally into the penile artery (7).

Suleman et al. described a modified approach to the DPNB and retrospectively analyzed data from 32 patients showing that an ultrasound-guided DPNB, compared to a landmark block, permits a reduction in the volume of local anesthetic and intraoperative opioids during GA. We based our approach on the one described by Suleman et al. (8).

Our modified technique has the advantage of having only one entry site on the skin of the patient and permits bilateral anesthesia of the dorsal nerves of the penis as well as the ventral part of the glans. Using the hydrodissection technique gives a circumferential spread of the local anesthetic with anesthesia of the entire penis.

It is safe because the procedure is ultrasound-guided in-plane and the needle only passes through the connective tissue, keeping distance from the corpora cavernosa and being opposite to the urethra.

Faraoni et al. compared the landmark and ultrasound-guided approach. In a randomized trial that enrolled 40 children who underwent circumcision under GA, they found in the ultrasound group a lower pain level in the first postoperative hour and a longer time to the first administration of analgesics, but with an increased procedural time. In this case, the approach was a sagittal puncture in the subpubic space, not penetrating the fascia of Buck, and the block was performed with ropivacaine 0.75% (12).

O’Sullivan et al. do not recommend the routine use of the DPNB because of a longer time in performing the block if compared to the landmark technique. The block was performed under GA, with the probe placed vertically above the pubis symphysis and the local anesthetic injected under the fascia of Scarpa but over the fascia of Buck with a bleb of local anesthetic at the base of the penis on the ventral side. No differences in intraoperative opioid consumption were noted, but only a reduction in the postoperative codeine use in the ultrasound-guided group (13).

A study conducted by Teunkens et al. found no differences between intraoperative and postoperative opioid consumption, with an increase in the induction time in the ultrasound-guided group and no benefit in the discharge time (9).

In the DPNB group, we performed the block under deep sedation maintaining the children always in spontaneous breathing. There was no need to induce GA and manage the airways with LMA or intubation. Our approach to the DPNB was novel, easy to perform, and with a minimal impact on the tissues of the children.

We used a local anesthetic with a rapid onset such as mepivacaine 1%, to allow surgery to begin immediately after the block was performed, associated with levobupivacaine 0.25%, to achieve a prolonged analgesic effect after the end of the procedure, minimizing the anesthetic concentration and reducing possible risks of toxicity (14, 15).

In the DPNB group, the target site was easily localized and the spread of the local anesthetic distinctly followed. Injection of the local anesthetic under the fascia of Buck appears to provide a circumferential spread of the local anesthetic with a more effective anesthesia, minimizing intraoperative opioid consumption, and improving postoperative analgesia.

There were no failures associated with the technique, as there was no increase in heart rate or blood pressure at the skin incision, and no need for airway management or mechanical ventilation. The complications rate was lower in the DPNB group, even if not statistically significant.

### Limitations

The main limitation of the study is that is only partially prospective, having a retrospective control group that might be a confounding factor. A prospective randomized trial with a larger study population could be useful to confirm the actual results.

## Conclusion

We found ultrasound-guided DPNB combined with sedation and spontaneous breathing is a time-saving, opioid-sparing, safe, and effective strategy for the management of intraoperative and postoperative pain in children undergoing circumcision.

## Data availability statement

The raw data supporting the conclusions of this article will be made available by the authors, without undue reservation.

## Ethics statement

The studies involving humans were approved by Ethics Committee of Friuli – Venezia Giulia. The studies were conducted in accordance with the local legislation and institutional requirements. Written informed consent for participation in this study was provided by the participants’ legal guardians/next of kin.

## Author contributions

BD: Conceptualization, Investigation, Methodology, Supervision, Writing – review & editing. FM: Conceptualization, Data curation, Investigation, Methodology, Writing – original draft, Writing – review & editing. ST: Investigation, Supervision, Writing – review & editing. DO: Formal analysis, Writing – review & editing. MC: Data curation, Investigation, Writing – original draft. NV: Investigation, Writing – original draft. LV: Methodology, Supervision, Writing – review & editing. SI: Investigation, Supervision, Writing – review & editing. TB: Methodology, Supervision, Validation, Writing – review & editing.
